# A Novel method of ensuring safe and accurate dilatation during percutaneous nephrolithotomy

**DOI:** 10.1590/S1677-5538.IBJU.2015.0007

**Published:** 2015

**Authors:** Tarun Javali, Amey Pathade, H. K. Nagaraj

**Affiliations:** 1 M.S. Ramaiah Hospital, Bangalore, India

**Keywords:** Nephrostomy, Percutaneous, Ureteroscopy

## Abstract

**Objective:**

To report our technique that helps locate the guidewire into the ureter enabling safe dilatation during PCNL.

**Materials and Methods:**

Cases in which the guidewire failed to pass into the ureter following successful puncture of the desired calyx were subjected to this technique. A second guidewire was passed through the outer sheath of a 9 Fr. metallic dilator cannula, passed over the first guidewire. The cannula and outer sheath were removed, followed by percutaneous passage of a 6/7.5 Fr ureteroscope between the two guidewires, monitoring its progress through both the endoscopic and fluoroscopic monitors. Once the stone was visualized in the calyx a guidewire was passed through the working channel and maneuvered past the stone into the pelvis and ureter under direct endoscopic vision. This was followed by routine tract dilatation.

**Results:**

This technique was employed in 85 out of 675 cases of PCNL carried out at our institute between Jan 2010 to June 2014. The mean time required for our technique, calculated from the point of introduction of the ureteroscope untill the successful passage of the guidewire down into the ureter was 95 seconds. There were no intraoperative or postoperative complications as a result of this technique. Guidewire could be successfully passed into the ureter in 82 out of 85 cases.

**Conclusions:**

Use of the ureteroscope introduced percutaneously through the puncture site in PCNL, is a safe and effective technique that helps in maneuvering the guidewire down into the ureter, which subsequently enables safe dilatation.

## INTRODUCTION

Establishing an effective and safe percutaneous access is the cornerstone in performing a successful and uneventful percutaneous nephrolithotomy. After successful puncture of the desired calyx, passage of the guidewire through the calyx into the pelvis and ureter provides the most secure position to proceed with tract dilatation. Failure to pass the guidewire through the punctured calyx down into the ureter may arise in some cases due to large stone bulk in the calyx or stone blocking the infundibulum. Dilatation over a guidewire coiled in a calyx vis-à-vis a guide wire successfully passed down a ureter is tenuous, with chances of slippage/dislodgement during dilatation, resulting in loss of access. The objective of the present study is to report our technique that helps locating the guidewire into the ureter enabling safe dilatation.

## MATERIALS AND METHODS

All patients underwent pre-operative evaluation with USG KUB, intravenous pyelography, urine culture and routine blood tests. PCNL was carried out in prone position under general anesthesia. Retrograde ureteric catheterization and fluoroscopy helped in defining the calyceal anatomy. Puncture was performed with an 18 G PCN puncture needle using the Bull's eye technique. Free flow of urine/saline confirmed successful puncture. A Terumo guidewire was then maneuvered to pass it through the punctured calyx down into the ureter. Cases in which this was successful proceeded with routine tract dilatation using telescopic metal dilators. In cases where the guidewire coiled in the calyx itself and failed to advance into the pelvis and ureter, we performed our technique (described below) to ensure safe location of the guidewire before proceeding with routine tract dilatation (Figures 1 and 2).

**Figure-1a:**
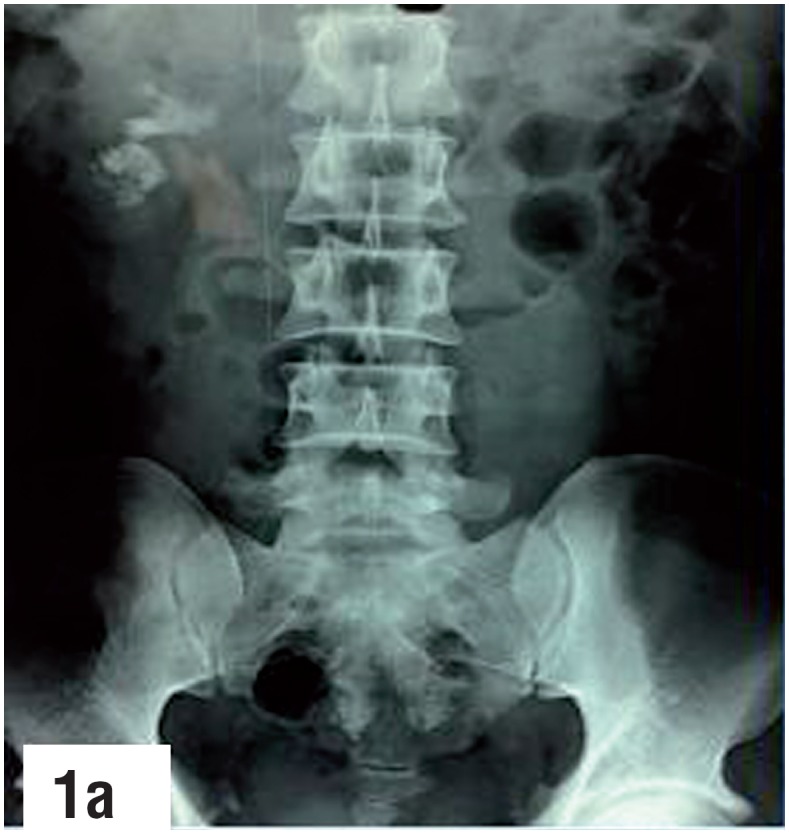
Plain X ray KUB showing inferior calyx and pelvic stone.

**Figure-1b:**
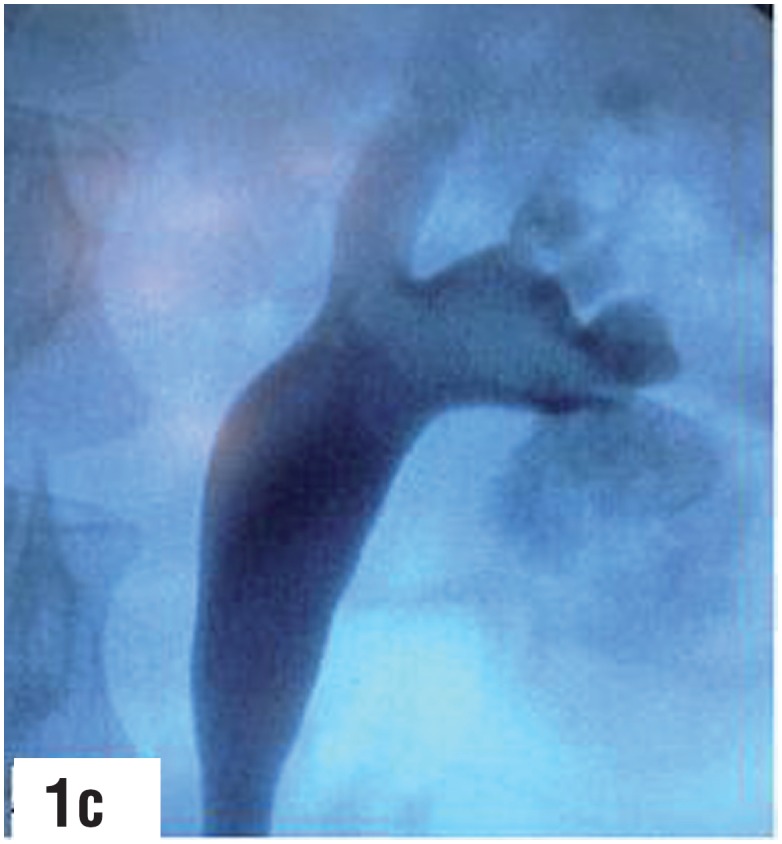
Retrograde Pyelogram in prone position, prior to puncture.

**Figure-1c:**
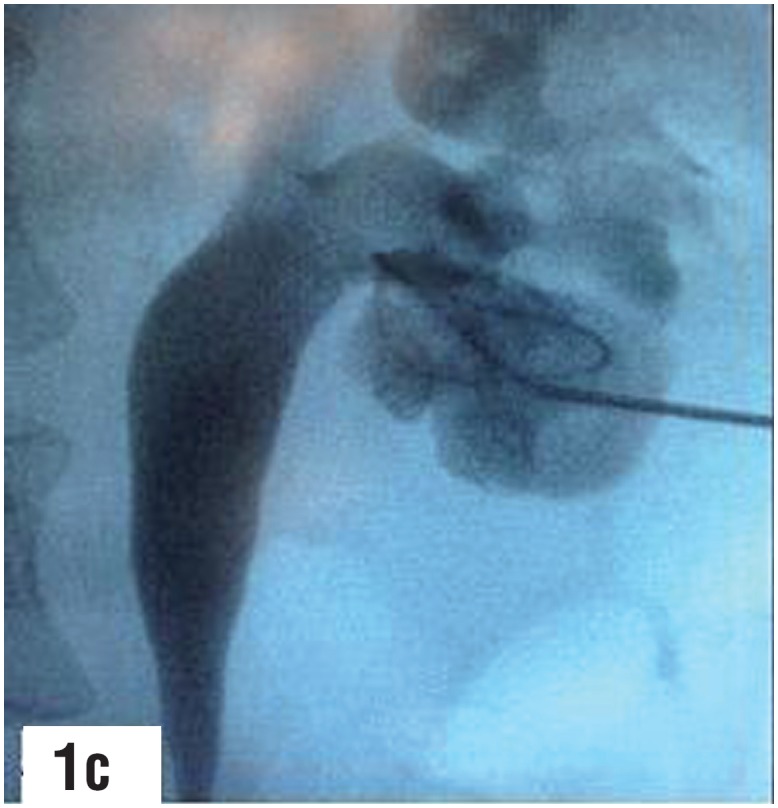
Lower calyx puncture, guidewire coiled in figure-1d - second guidewire coiled into the lower calyx lower calyx. guidewire could not be located beyond the through the outer sheath of the puncture cannula. infundibulum of the punctured calyx. Dilatation at this stage would be tenuous with the possibility of slippage of guidewire resulting in loss of access.

**Figure-1d:**
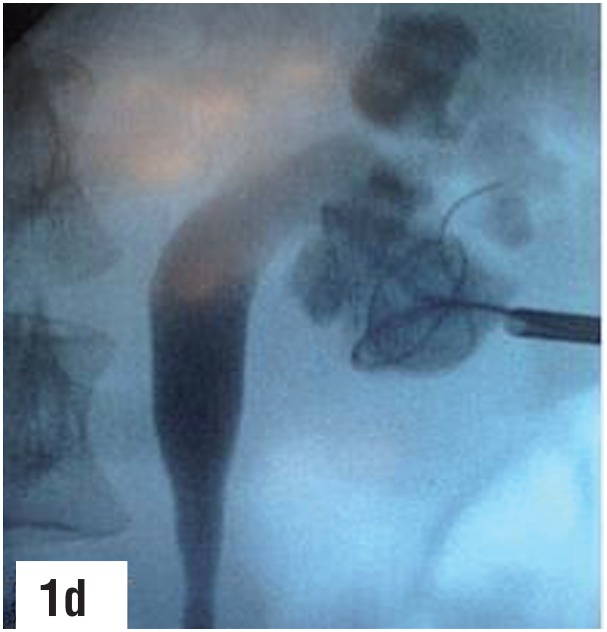
Second guidewire coiled into the lower calyx through the outer sheath of the puncture cannula.

**Figure-1e:**
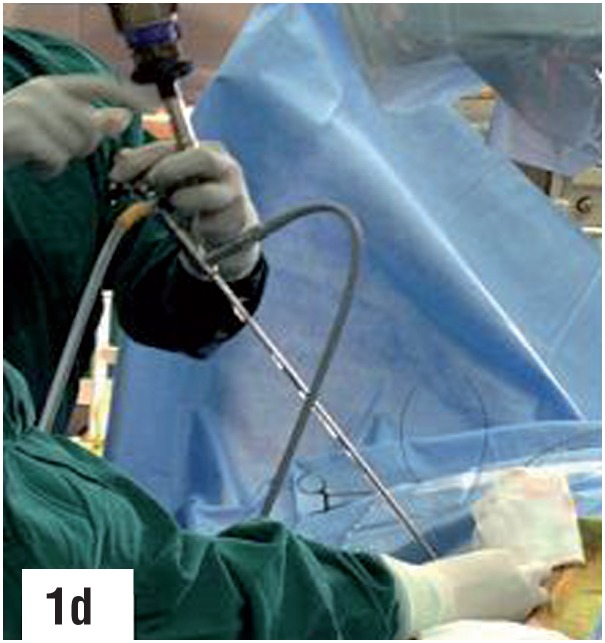
Ureteroscope being passed percutaneously through the puncture site.

**Figure-1f:**
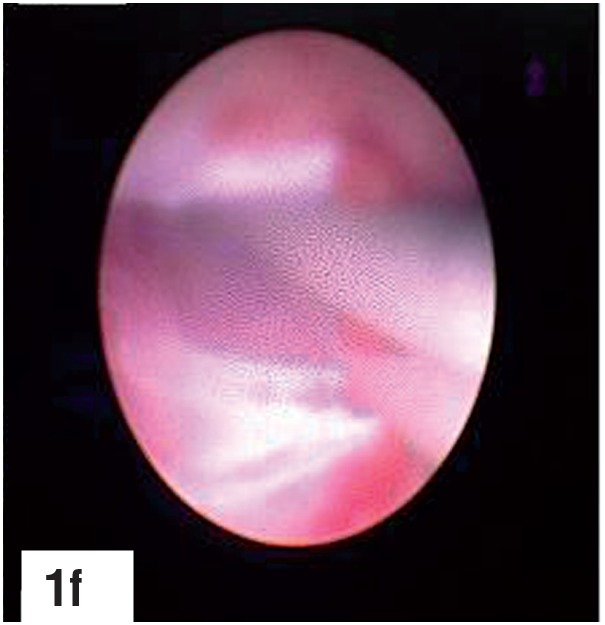
Ureteroscope passed between the two guidewires.

**Figure-2a:**
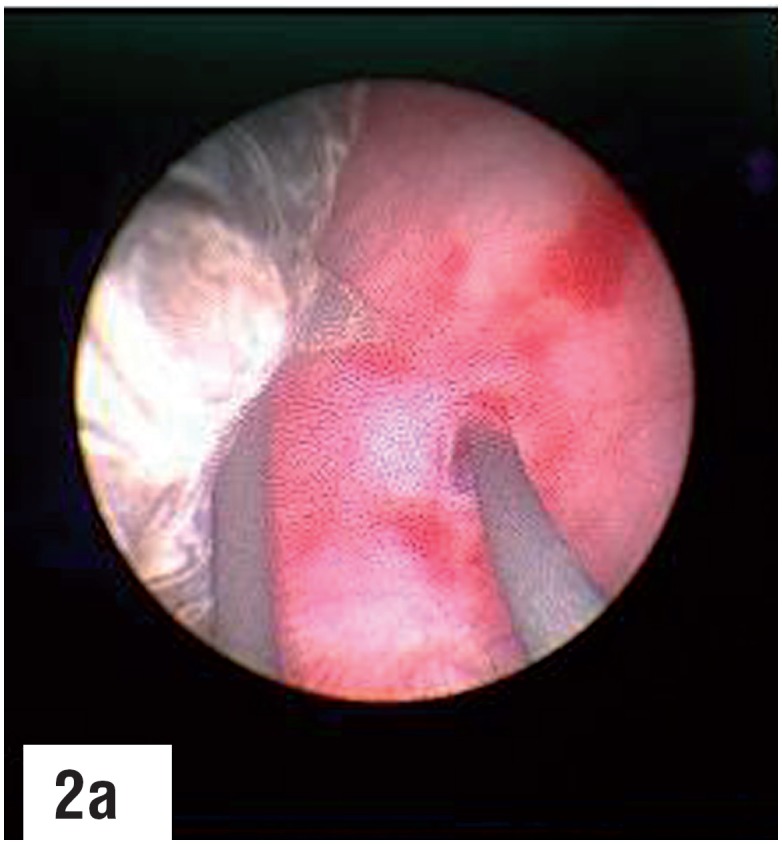
The progress of the ureteroscope is monitored simultaneously through both the endoscopic and fluoroscopic monitors untill the stone is visualized. Third guidewire is passed through the working channel of the ureteroscope and is maneuvered to bypass the stone under vision. note that the previously passed guidewire, which was assumed to be coiled in the calyx fluoroscopically, had in fact perforated the mucosa. Dilatation over this guidewire would have been hazardous.

**Figure-2b:**
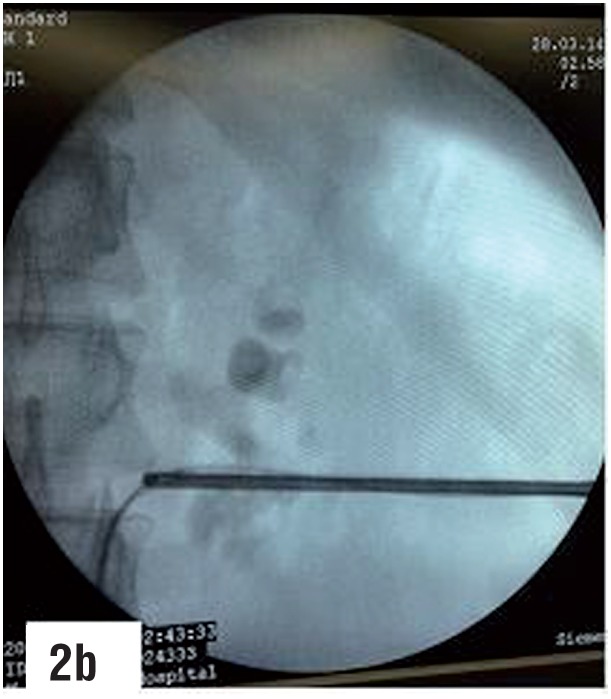
Guidewire has been successfully passed down into the ureter.

**Figure-2c:**
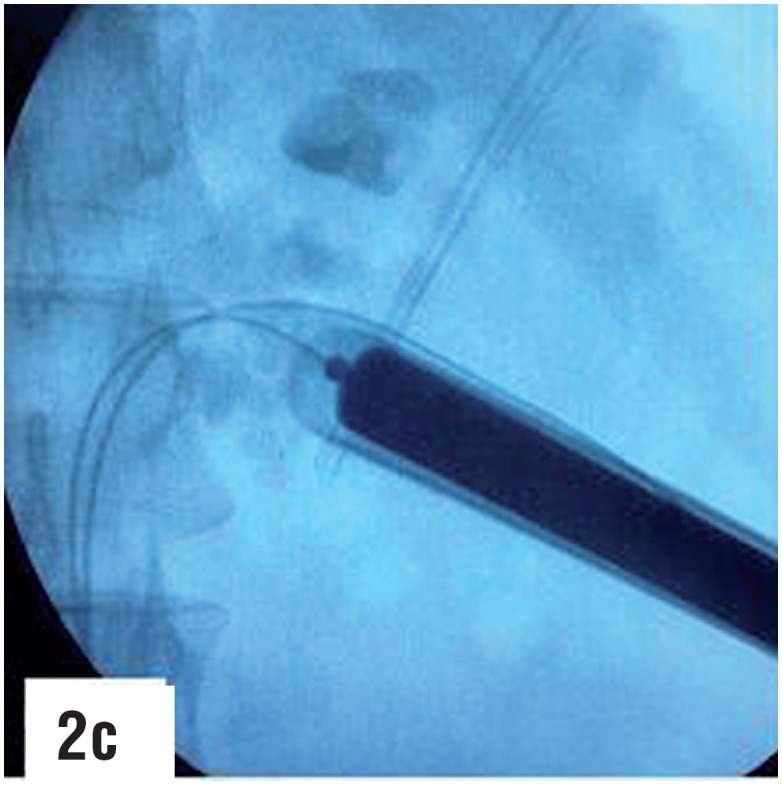
Routine dilatation is carried out over the newly passed guidewire to enable passage of amplatz sheath of desired size.

A 9 Fr. metallic dilator cannula with outer sheath was passed over the guidewire under fluoroscopic guidance into the calyx. The outer sheath was advanced into the calyx followed by removal of the inner cannula. A second guidewire was passed through the outer sheath, which was then withdrawn. An 6/7.5 Fr. semirigid ureteroscope was passed percutaneously through the puncture wound between the two guidewires, monitoring its gradual progress via both the endoscopic as well as the fluoroscopic monitors, till the semirigid ureteroscope entered the correct calyx and the stone was visualized. The two guidewires served as “safety wires”, comparable to “tramtrack” or “railway lines”, between which the semirigid ureteroscope was gradually passed, taking care that the two guidewires were always in vision. The ureteroscope was not passed over one of the guidewires or through the outer sheath as the former may result in buckling of the guidewire and the later may hinder the maneuverability of the semirigid ureteroscope. A third guidewire was then passed through the working channel of the semirigid ureteroscope, and under direct vision, maneuvered by the side of the stone, through the infundibulum, into the pelvis and ureter. The first two guidewires were then removed and routine tract dilatation was then carried out over the guidewire that was located into the ureter, after withdrawing the semirigid ureteroscope. The rest of the procedure was then carried out in the usual manner.

## RESULTS

Between January 2010 to June 2014, a total of 675 PCNLs were carried out at our institute. Consultants or final year urology residents under supervision performed all procedures. We had to resort to our technique in 85 of these cases (12.5%). Table-1 gives the patient data analysis of these 85 cases. The mean time required for our technique was calculated from the point of introduction of the semirigid ureteroscope untill the successful passage of the guidewire down into the ureter. In three patients, with calyceal and tightly impacted pelviureteric junction stones, after puncture of the lower calyx, the guidewire could not be passed into the ureter and was instead coiled into the superior calyx. In all these three patients, initial attempt with the traditional approach resulted in failure to advance the guidewire beyond the punctured calyx itself. In the remaining 82 cases, the guidewire could be successfully moved out of the punctured calyx into the ureter. There were no intraoperative or postoperative complications in the 85 patients in whom this technique was employed.

**Table 1:** Patient Data.

Characteristics		Data
Total number of PCNLs		675
No. in which our technique was used; (%)		85 (12.5%)
Mean age; (range)		45.5 yrs (16-71 yrs)
Previous open surgery		12
**Puncture**		
	Superior calyx	2
	Middle calyx	32
	Lower calyx	51
Mean time taken to park the guidewire into the ureter with our technique;(range)		95 secs (45- 135secs)
Mean fluoroscopy time from insertion of ureteroscope to passage of guidewire into ureter		8.8secs (6-14secs)
Total time from puncture to sheath placement		210 secs (125-280 secs)
Mean fuoroscopy time from puncture to insertion of sheath		15.5 secs (12-22 secs)
Complications		nil

## DISCUSSION

The three most important steps in performing PCNL are:

1. Accurate puncture
2. Passage and location of the guidewire into the pelvicalyceal system in such a way as to prevent slippage (the most ideal being passage of guidewire into the ureter)
3. Dilatation

Current literature on PCNL emphasizes a lot on steps 1 and 3 (1–7). Our technique ensures location of the guidewire into the ureter after a successful puncture to enable safe dilatation and highlights the importance of step 2. This was a single arm, single institution retrospective series of an alternative method of placing a guidewire down the ureter during percutaneous access, in instances where fluoroscopic placement was unsuccessful.

Various devices and alternative techniques have been described in literature in order to obtain a better puncture and to pass a perfect guidewire (8). Retrograde nephrostomy by Lawson's procedure was reported in 1980. Grass et al. (9) described flexible ureteroscopy for assisting antegrade percutaneous renal access under fluoroscopic control in difficult cases. Bader et al. (1) reported their experience with the “all–seeing needle” as an optical puncture system in PCNL for achieving optimal renal access. The micro-optics of 0.9mm and 0.6mm diameter with integrated light lead was passed through the working channel of a 4.85 Fr. access needle, which was connected to an irrigation system for better intraoperative view. By this technique the punctured calyces and calculi could be visualized in all 15 patients prior to placement of guidewire and tract dilatation. Chen et al. (10) described a novel device called ‘sonic flashlight’ to visualize and guide the needle during renal access. This technique is a renal-time tomographic reflection that generates a virtual anatomically scaled image. The demonstration of the sonographic image appears from the tip of the transducer. The authors claimed that this technique could facilitate safe renal access for complicated cases. Other techniques described include a computer assisted gantry system (4) and mobile augmented reality for computer assisted PCNL (5).

Our technique is easy to learn and inexpensive as no sophisticated instruments are required. Furthermore, no complications related to our technique were observed, reiterating that it is safe to instrument a newly created tract into the kidney.

## CONCLUSION

Use of the ureteroscope passed percutaneously through the puncture site in PCNL is a safe and effective technique that helps in maneuvering the guidewire down into the ureter, which subsequently enables safe dilatation.

## ABBREVIATIONS

USG KUB: = Ultrasonogram kidney-ureter-bladder
PCNL: = Percutaneous Nephrolithotomy
PCN: = Percutaneous Nephrostomy
